# 

**Published:** 1894-04

**Authors:** 


### CONTENTS.

***EDITORIALS.***

PAGE

The Plate that appears as a Frontispiece, . . . . . . . 97

The Habit of Generalization, . . . . . . . . . . 97

***MISCELLANEOUS.***

Clinical Observations—Bismuth in Cholera Infantum. Ad. Lippe, M. D., . . . . . . . . . . . . . . 100

Renal Colic. A Quackenbush, M. D., . . . . . . . 102

Selected Eye Symptoms. Mahlon Preston, M. D., . . . . . 104

The Editorial in the February Number. C. Carleton Smith, M. D., 106

Gleanings. F. H. Lutze, M. D., . . . . . . . . . 108

The Homœopathic Club of Denver, Col., . . . . . . . 119

Three Cases of Intermittent Fever and how they were treated. C. M. Boger, M. D., . . . . . . . . . . . . 120

A Nux Case, "Model Cure." G. J. Waggoner, M. D., . . . . 122

***BOOK NOTICES.***

A Standard Dictionary of the English Language, . . . . . 122

Essentials of Homœopathic Materia Medica. By W. A. Dewey, M. D., . . . . . . . . . . . . . 125

The Physician's Wife. By Ellen M. Firebaugh, . . . . . 126

Photographic Panorama of the World's Fair, . . . . . . 126

Scientific American. Architects' and Builders' Edition, . . . 126

Longevity. By Archer E. Atkinson, M. D., . . . . . . 127

An Anatomist's Anomaly Blank, . . . . . . . . . 127

***NOTES AND NOTICES.***

Annual Reunion Alumni Association—Frederick Stearns & Co.—Dr. Millie J. Chapman—John M. Scudder, M. D.—The North Indiana and Southern Michigan Medical Association, . . . 128

